# Occult Pneumonia in an Immunocompetent Adult With Initially Normal Imaging and Laboratory Results: A Case Report

**DOI:** 10.7759/cureus.93540

**Published:** 2025-09-30

**Authors:** Mohamed Elnakib

**Affiliations:** 1 Internal Medicine, Umm Al-Qura Medical Center, Makkah, SAU

**Keywords:** acute pneumonia, chest infection, diagnosis, fever, headache, late diagnosis, physical diagnosis

## Abstract

We present the case of a 38-year-old immunocompetent man who developed pneumonia despite initially normal laboratory tests and radiological imaging. The patient experienced persistent fever, headache, and myalgia but had no cough or respiratory distress. Repeat clinical evaluation revealed new localized lung findings that prompted further investigation and treatment. This case highlights the importance of repeated assessment, careful physical examination, patient-centered decision-making, and clinical vigilance in the management of atypical presentations of common infections.

## Introduction

Pneumonia remains a major cause of morbidity and mortality worldwide, with the World Health Organization ranking it among the leading infectious causes of death [1,2]. In adults, the clinical diagnosis is usually guided by the presence of fever, cough, dyspnea, chest pain, and localized findings on auscultation, together with supporting laboratory and radiological evidence [3,4]. However, the presentation is not always typical. Occult pneumonia describes a subset of cases where initial clinical findings, blood tests, or imaging are inconclusive, delaying diagnosis and treatment [5,6]. This atypical presentation is particularly challenging in immunocompetent adults, where pneumonia is not always considered early in the differential diagnosis [7].

Case fatality rates for community-acquired pneumonia vary by region and patient population, ranging from less than 5% in otherwise healthy adults to more than 15% in hospitalized or critically ill patients [8,9]. Delayed recognition of pneumonia due to atypical or occult presentations contributes to worse outcomes, especially when empirical treatment is deferred [3,10]. Previous reports have emphasized that even a normal chest radiograph or laboratory profile does not fully exclude early pneumonia [4,5].

We report the case of an immunocompetent adult who presented with persistent fever and systemic symptoms but minimal respiratory findings and initially normal investigations. The diagnostic challenge was compounded by inconclusive imaging and limited microbiological data. This case highlights the importance of maintaining a high index of suspicion, repeating clinical evaluation, and considering pneumonia even when initial tests appear unrevealing [1,2].

## Case presentation

A previously healthy 38-year-old male patient presented with a five-day history of high-grade fever, generalized myalgia, and malaise [2,3]. He denied cough, sputum production, chest pain, shortness of breath, or gastrointestinal and urinary symptoms. There was no history of tuberculosis, chronic lung disease, or recent animal exposure [1,4]. He was a non-smoker with no significant occupational exposures [1].

At an outside hospital, he was initially assessed as having a possible viral illness or fever of unknown origin [4]. Baseline blood tests, including complete blood count (CBC), renal function, and liver enzymes, were within normal limits. A chest computed tomography (CT) scan was obtained early because of persistent fever despite supportive care and showed no pulmonary consolidation [4,5]. He was discharged with symptomatic treatment.

Three days later, he presented to our emergency department with ongoing fever and worsening fatigue [2]. Upon examination, he was febrile at 39.2 °C, had a heart rate of 110 bpm, a blood pressure of 120/70 mmHg, and an oxygen saturation of 97% on room air. Chest examination revealed faint inspiratory crackles localized to the right lower lung zone, which were not documented during the first visit [5]. Neurological and abdominal examinations were unremarkable.

Laboratory investigations demonstrated a white blood cell count of 11.8 × 10^9^/L (reference: 4-10 × 10^9^/L), C-reactive protein level of 112 mg/L (reference: <5 mg/L), and erythrocyte sedimentation rate of 55 mm/h [2,6]. Renal and liver function tests remained normal. Blood cultures were negative, and sputum could not be obtained. No urinary antigen or polymerase chain reaction (PCR)-based pathogen testing was available [9].

**Figure 1:**
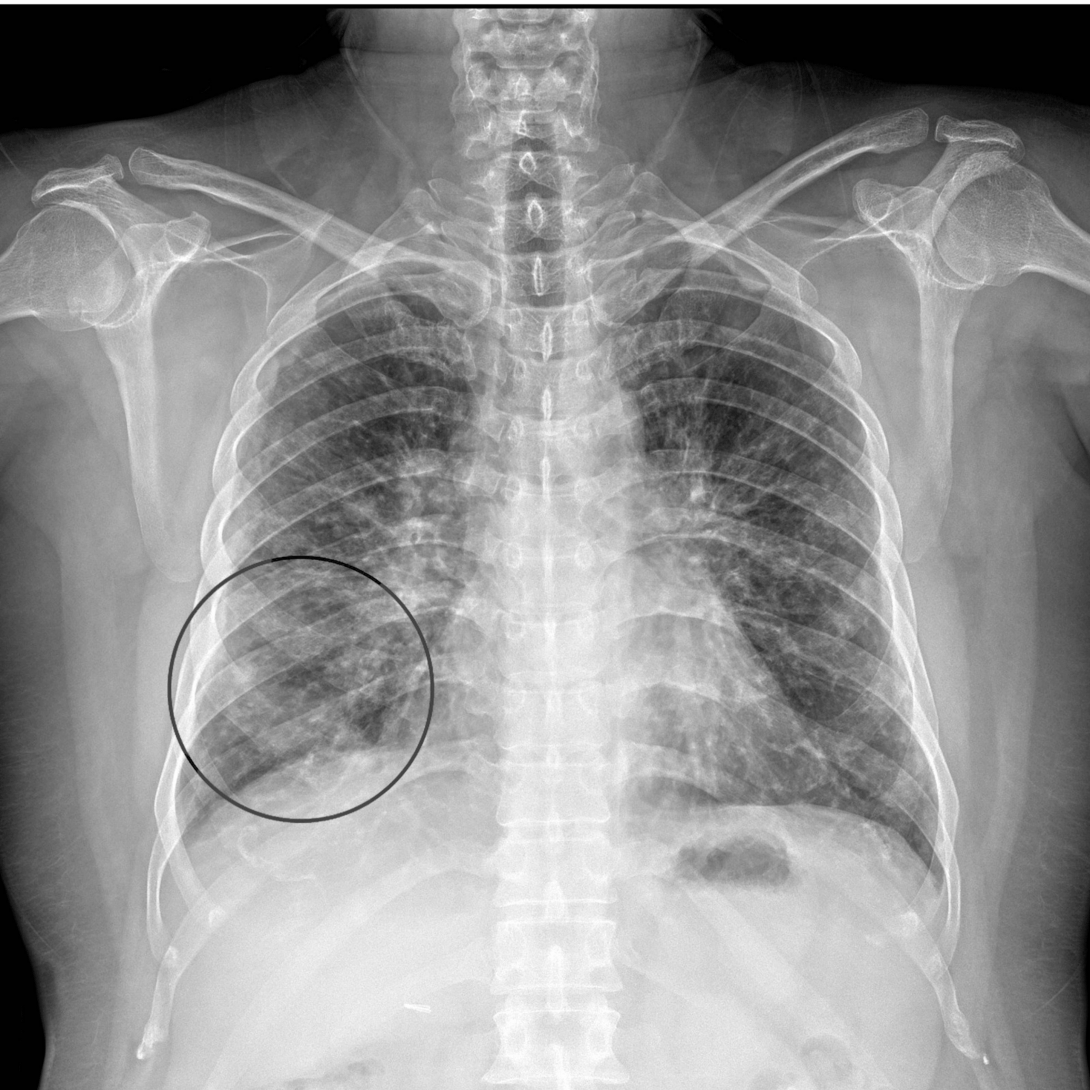
Frontal chest radiograph (April 12, 2025) showing patchy consolidation in the right lower lung zone, consistent with evolving pneumonia.

A chest radiograph obtained at this visit revealed a patchy opacity in the right lower zone (Figure 1) [6]. The radiologist’s interpretation favored early lobar pneumonia [6,9].

Based on these findings, the patient was started on empiric intravenous (IV) ceftriaxone and clarithromycin, in accordance with local guidelines for community-acquired pneumonia [2,6]. His fever subsided within 48 hours, and his clinical condition improved. After four days, he was transitioned to oral amoxicillin-clavulanate to complete a seven-day course [2].

The patient was discharged in stable condition, and at follow-up two weeks later, he reported complete resolution of symptoms [2,6]. The overall clinical course is summarized in Table 1 [5,6].

**Table 1:** Timeline of clinical course. CBC: Complete blood count; CT: Computed tomography; IV: Intravenous

Timeline (relative to presentation)	Date (2025)	Clinical events
Day -14 to -8	Mar 29 - Apr 4	Onset of fever, myalgia, and headache. Initial evaluation at outside hospital with normal labs and chest CT.
Day 0	Apr 12	Presented to our hospital. Exam revealed right basal crackles. Repeat CBC showed leukocytosis and elevated inflammatory markers. Frontal chest X-ray demonstrated right lower zone opacity.
Day 0 to 2	Apr 12 - 14	Empiric IV ceftriaxone and clarithromycin initiated. Fever subsided within 48 hours with marked clinical improvement.
Day 3 to 7	Apr 15 - 19	Continued inpatient recovery. Transitioned to oral amoxicillin-clavulanate. Discharged in stable condition.
Day 7 to 14	Apr 19 - 26	Outpatient follow-up confirmed complete resolution of symptoms.

## Discussion

This case illustrates the diagnostic challenge of pneumonia presenting with persistent fever but minimal respiratory symptoms in an immunocompetent adult [1,2,6]. While classic community-acquired pneumonia typically presents with cough, dyspnea, pleuritic chest pain, and fever [1,2], atypical or occult presentations can occur and delay recognition [4,5]. Occult pneumonia is defined as radiographic evidence of pulmonary infection in patients lacking prominent respiratory symptoms [4]. It is well-described in children [10] but rarely reported in adults, particularly when initial laboratory tests and imaging are normal [4-6].

Early investigations, including a chest CT and baseline labs, were unremarkable in this patient [4,5]. This highlights a known limitation: early-stage pneumonia may not yet produce radiographic changes or marked laboratory abnormalities [5,6]. Repeat clinical assessment, including focused auscultation, was key to detecting new focal findings that prompted a chest X-ray demonstrating evolving right lower-zone consolidation [6,9].

Differential diagnoses initially considered included viral illness, tuberculosis, zoonotic infections (e.g., Q fever, brucellosis, psittacosis), autoimmune/inflammatory conditions, and occult malignancy [3,4]. The emergence of localized crackles, elevated inflammatory markers, and rapid response to empiric antibiotics supported bacterial pneumonia as the leading diagnosis [2,6].

Microbiological testing was limited by the absence of sputum production, negative blood cultures, and the unavailability of urinary antigen or PCR tests for *Legionella*, S*treptococcus pneumoniae*, and viral pathogens [9]. These limitations reflect real-world practice in resource-constrained settings but do not negate the importance of repeated clinical assessment and empiric management [2,6].

Management followed local and international guidelines for community-acquired pneumonia [1,2,6]. Empiric ceftriaxone and clarithromycin were started to cover typical and atypical bacterial pathogens, followed by step-down oral therapy [2,6]. The patient’s rapid clinical improvement emphasizes the importance of timely empiric treatment once new findings emerge [2,6].

Ethical and communication aspects were central to patient care. Explaining the rationale for repeat investigations and empiric antibiotic therapy addressed the patient’s frustration, supported informed decision-making, and enhanced trust and adherence [2,6].

This case is instructive for clinicians: persistent fever despite initially normal investigations should prompt repeat evaluation, careful physical examination, and consideration of pneumonia even in the absence of cough or radiographic abnormalities [1,2,4,6]. Clinicians should remain vigilant for atypical presentations and reassess patients proactively, as early recognition and treatment are critical for favorable outcomes [2,6,9].

## Conclusions

Pneumonia can present atypically in immunocompetent adults, with persistent fever and systemic symptoms but minimal or absent respiratory signs and initially normal laboratory tests and imaging. This case highlights the importance of repeated clinical assessment, careful auscultation, and vigilance for evolving findings. Prompt empiric antibiotic therapy guided by new clinical signs can lead to rapid recovery. Clinicians should consider pneumonia in the differential diagnosis of persistent fever, even when early investigations appear unrevealing, and communicate clearly with patients to support adherence and informed decision-making.
